# Could music potentially serve as a functional alternative to alcohol consumption? The importance of music motives among drinking and non-drinking adolescents

**DOI:** 10.1556/JBA.3.2014.4.3

**Published:** 2014-12-18

**Authors:** ANNA JONKER, EMMANUEL KUNTSCHE

**Affiliations:** ^1^Behavioural Science Institute, University of Nijmegen, the Netherlands; ^2^Addiction Switzerland, Research Institute, Lausanne

**Keywords:** music motives, drinking motives, alcohol use, adolescents

## Abstract

*Background and aims:* This study investigated whether adolescents who drink and those who are teetotal differ in the link between music motives and health-related outcomes (life satisfaction, self-rated health, school pressure, somatic complaints, depressed and aggressive mood, physical powerlessness, frequency of being bullied and bullying others and evenings spent out with friends). It also looked at whether associations between music motives and health-related outcomes remained significant when drinking motives were included among drinkers. *Methods:* Confirmatory factor analysis and structural equation models were estimated based on data from 4,481 adolescents from Switzerland (mean age 14.5, *SD* = 0.9). *Results:* It was confirmed that the four music motives and the four drinking motives obtained by crossing the valence (positive–negative) and the source (internal–external) of expected change in affect form distinct dimensions (i.e. the 8-factor model best fitted the data). Drinkers and non-drinkers differed in the various links between music motives and health-related outcomes. For example, almost all the links between conformity music motives and the health-related outcomes were significant for non-drinkers but not for drinkers. Enhancement music motives, by contrast, were often significant for drinkers but not for non-drinkers. Coping music motives were significant among both drinkers and non-drinkers. These links were basically unchanged when drinking motives were taken into account. *Discussion and conclusions:* This study indicates that music serves important functions in the lives of adolescents, even among those who use alcohol for different motives. This makes listening to music a promising potential alternative to alcohol use.

## INTRODUCTION

### Functions of music

During the second decade of life, substance use (including alcohol), and use of media (including listening to music) become more and more common (Currie et al., 2012; Roberts & Christenson, 2001). For example, in response to the question regarding what they would take to a desert island, 40% of adolescents in 7^th^ grade indicated music as their first choice; in 11^th^ grade this number increased to more than half (Roberts & Christenson, 2001). There are several functions that music can serve among adolescents, such as bringing them together, regulating their emotions and optimizing their level of arousal and self-reflection on hopes, wishes and concerns (Laiho, 2004). One of the negative consequences of listening to music is hearing impairment. In her meta-analysis, Daniel (2007) shows that many adolescents are exposed to loud music via headphones and car sound systems, during loud concerts and in night clubs. Another study demonstrated that alcohol use may be instigated by the drinking-related lyrics in songs (Engels, Slettenhaar, ter Bogt & Scholte, 2011).

### Expected affective change: the motivational model

To investigate what motivates adolescents to listen to music based on the affective change that individuals strive to obtain, Kuntsche, Le Mével and Berson (in press) developed and validated the Motives for Listening to Music Questionnaire (MLMQ). The MLMQ was inspired by the assumptions of the Motivational Model of Alcohol Use by Cox and Klinger (1988, 1990). This model assumes that drinking motives are characterized by two dimensions: the valence (positive or negative) and the source (internal or external). Individuals may drink to obtain a positive outcome or to avoid a negative outcome. In addition, individuals may achieve internal rewards (e.g. change their internal emotional state) or external rewards (e.g. social acceptance) by drinking alcohol. By crossing the two dimensions, Cooper (1994) obtained the four motive factors: “enhancement” (internal, positive), “social” (external, positive), “coping” (internal, negative) and “conformity” (external, negative).

Although originally developed to understand alcohol use, these four motivational factors have been used to investigate other domains of human behaviour such as gambling (Stewart & Zack, 2008) or sexual risk-taking behaviour (Cooper, Shapiro & Powers, 1998). In the MLMQ, the four-factor structure of music motives was confirmed, as were the hypothesized associations between coping motives and health-related outcomes (somatic complaints, aggressive and depressed mood, school pressure, low life satisfaction, etc.), between social motives and peer-related activities (spending evenings with friends, bullying others, etc.), and between conformity motives and being depressed and a victim of bullying (Kuntsche, Le Mével & Berson, in press). Focusing on the affective change adolescents strive to obtain by listening to music, the MLMQ has the advantage that it does not depend on the genre of music the adolescents are listening to, e.g. according to personal taste either classical, folk, rap, rock or another musical genre may be used to cheer up when in a bad mood.

### Study aims

A strict comparison between what motivates adolescents to drink alcohol and what motivates them to listen to music, and the similarities and differences between these motivations in respect to health-related outcomes is still lacking. Therefore, this study investigates the links between music motives and health-related outcomes among drinkers and non-drinkers when drinking motives are taken into account. Given the fact that drinking motives can only be assessed among drinkers, our first aim was to test differences in music motives between drinkers and non-drinkers. Before we could test the link between drinking motives and music motives, we had to be sure that the two concepts form distinct dimensions. Therefore, our second aim was to test the hypothesis that drinking motives and music motives form eight distinct dimensions according to the behaviour – listening to music and drinking – and according to the four hypothesized motivational dimensions by crossing type of reinforcement (positive vs. negative) and source of expected effects (internal vs. external). Our third aim was to test whether music motives are still important among drinkers when drinking motives are taken into account. Our hypothesis was that music motives predict the different health-related outcomes in adolescence among drinkers, even when drinking motives are taken into account. This was investigated by testing the link between music motives and the outcomes in adolescence, and then adding drinking motives. Before we could do this, we tested differences in the effects of music motives on the outcomes in adolescence between drinkers and non-drinkers, since drinking motives can only be assessed among drinkers.

If music motives remain important among drinkers, this has implications for prevention. If this turns out to be the case, prevention programs should not only target the specific drinking motives, but could potentially also offer music to adolescents as a functional alternative to alcohol use to obtain a similar effect.

## METHODS

### Study design

Data were used from the Swiss participation (www.HBSC.ch) in the international survey “Health and Behaviour in School-Aged Children” (www.HBSC.org). Within a random cluster sampling design, 5^th^ to 9^th^ grade state school classes were randomly selected proportionate to the size of the participating Swiss cantons. The response rate was 88%. Between January and April 2010, pupils were given 45 minutes to voluntarily complete the questionnaire on their own in the classroom setting.

### Sample and missing values

The sampling was based on lists of Swiss schools from 5^th^ to 9^th^ grade, compiled by the Swiss Federal Statistical Office. The 5^th^ to 7^th^ graders received a shortened version of the questionnaire for comprehension and ethical reasons, in which music motives and drinking motives were not included. The original sample comprised 4,644 8^th^ and 9^th^ grade adolescents from state schools in Switzerland. The participants who failed to answer all the items on one or more music dimensions (*n* = 121; 2.7%) or had inconsistent answers on alcohol use and drinking motive questions (*n* = 2; >0.1%) were excluded from further analysis. The participants with missing values on age (*n* = 27; 0.6%) and gender (*n* = 19; 0.4%) were also excluded. The analysed data consists of 4,481 adolescents (mean age = 14.5, *SD* = 0.9; age range 12 to 18 years; 48.8% male). Of the adolescents 2,720 (60.7%) were drinkers and 1,761 (39.3%) were non-drinkers.

### Measures

#### Drinking motives

Motives for drinking were assessed with DMQ-R SF (Kuntsche & Kuntsche, 2009), which consists of 12 items to measure the four dimensions “Enhancement”, “Social”, “Coping” and “Conformity”. The answer categories were “almost never/never” (coded as 1), “some of the time” (2), “half of the time” (3), “most of the time” (4) and “almost always/always” (5).

#### Listening to music motives

Motives for listening to music were assessed with MLMQ (Kuntsche et al., in press). Based on the DMQ-R SF, the number of items, dimensions and response options were identical (for details, see Appendix).

#### Life satisfaction

Using the Cantril ladder (1965), adolescents had to rate their life satisfaction on a ten-point scale ranging from 0 (“worst possible life”) to 10 (“best possible life”) (Kuntsche & Gmel, 2004).

#### School pressure

School pressure measured the global amount of pressure or stress caused by the demands of schoolwork and homework. Answer categories were “not at all” (coded as 1), “a little” (2), “some” (3) and “a lot” (4).

#### Self-rated health

Adolescents were asked “Would you say your health is…”, followed by the answer categories “poor” (coded as 1), “fair” (2), “good” (3) and “excellent” (4).

#### Health complaints

The HBSC Symptom Checklist (HBSC-SCL: Haugland & Wold, 2001) contains items on somatic complaints (“headache”, “stomach ache”, “backache”, a = .58), aggressive mood (“irritability and bad temper”, “felt annoyed and angry”, a = .76), depressed mood (feeling “low”, “nervous”, “anxious or worried”, a = .72) and physical powerlessness (“difficulties getting to sleep”, “feeling dizzy”, “feeling tired”, a = .55). Answer categories were coded to represent monthly frequencies: “rarely or never” (coded as 0), “about every month” (1), “about every week” (4.5), “more than once a week” (9) and “about every day” (30).

#### Frequency of being bullied

Based on the work of Olweus (1994), adolescents had to indicate how often they had been bullied at school in the previous couple of months. Answer categories were “did not happen” (coded as 0), “once or twice” (1.5), “2 or 3 times a month” (5), “about once a week” (9) and “several times a week” (18).

#### Frequency of bullying others

Adolescents were asked to indicate how often they had taken part in bullying other student(s) at school in the previous couple of months. Answer categories were identical to the ones for being bullied.

#### Evenings spent with friends

Adolescents were asked to indicate how many evenings per week they usually spend out with their friends. The eight answer categories ranged from “no evenings” (codes as 0) to “7 evenings” (7).

### Statistical analysis

All analyses were conducted in the statistical software Mplus (Muthén & Muthén, 2010). To account for the dependency and non-normal distribution of observations (e.g. adolescents nested within school classes), we used the Mplus complex sampling option and the MLR estimator (Maximum Likelihood Robust).

First, we used logistic regression to test whether drinkers (*n* = 2,270; 60.7%, coded as 1) differ from non-drinkers (*n* = 1,761; 39.3%, coded as 0) in the four music motive dimensions (i.e. enhancement, social, coping and conformity). We adjusted this analysis for age and gender effects.

Second, to confirm that drinking motives and music motives form different dimensions, we used confirmatory factor analysis. Since drinking motives cannot be assessed among non-drinkers, the sample comprised 2,720 adolescents (60.7%) who had drunk alcohol at least once in the previous 12 months. We compared the model fit of a two-dimensional structure, a four-dimensional structure and an eight-dimensional structure. In the two-dimensional solution, all drinking motive items were loaded on one factor and all music motive items on another; the four-dimensional structure contained the dimensions enhancement, social, conformity and coping, that consisted of both the items of drinking motives and music motives; and the eight-dimensional structure contained the dimensions enhancement drinking motives, enhancement music motives, social drinking motives, social music motives, coping drinking motives, coping music motives, conformity drinking motives and conformity music motives. We used the CFI (Comparative Fit Index), the TLI (Tucker-Lewis Index), the RMSEA (Root Mean Square Error of Approximation) and the SRMR (Standardized Root Mean Square Residual) to evaluate the model. For an acceptable fit, the CFI and TLI values should both be .95 or higher, while the RMSEA and the SRMR values should both be .08 or lower (Schreiber, Nora, Stage, Barlow & King, 2006).

Third, we estimated multivariate linear structural equation models to test the expected association of music motives and several health-related outcomes in adolescence. The models were estimated separately, with the four music dimensions as independent variables, gender and age as control variables, and a particular outcome in adolescence as a dependent variable (i.e. life satisfaction, school pressure, self-rated health, somatic complaints, aggressive mood, depressed mood, physical powerlessness, frequency of being bullied, frequency of bullying and evenings spent with friends). To test differences in the effects of music motives on the outcomes in adolescence between drinkers and non-drinkers, the models were estimated separately for drinkers (*n* = 2,720; 60.7%) and non-drinkers (*n* = 1,761; 39.3%). To reduce the complexity of these very large models, summary scores of the different motive dimensions were used.

Subsequently, among drinkers, we investigated whether the expected association between music motives and several outcomes in adolescence remains significant when drinking motives are included simultaneously in this model.

### Ethics

Anonymity and privacy were guaranteed by asking pupils not to write their names on the questionnaires and to put the questionnaires in an unmarked envelope after completion and seal it. The study was approved by the Human Research Ethics Committee (Canton of Vaud Protocol no. 173/09). In each participating canton and school, the educational authorities and head teachers both gave permission to conduct the HBSC survey.

## RESULTS

### Step 1: Logistic regression

Drinkers were less likely to indicate high levels of enhancement music motives and high levels of conformity music motives than non-drinkers (Table 1). They were more likely to indicate high levels of social music motives than non-drinkers.

**Table 1.** Odds ratios and 95% confidence interval (in brackets) to predict the differences between music motives and being a drinker (coded as 1) or a non-drinker (coded as 0) adjusted for age and gender effects

	Odds ratio
Enhancement motives	0.861***	(0.782–0.948)
Social motives	2.068***	(1.930–2.215)
Coping motives	1.037	(0.970–1.108)
Conformity motives	0.648***	(0.619–0.756)
Age	1.441***	(1.328–1.564)
Gender (male = 1)	1.556***	(1.338–1.811)

*Note:* *** *P* < 0.001.

### Step 2: Confirmatory factor analysis

The two-dimensional structure, i.e. general drinking motivation (12 items) vs. general music motivation (12 items) and the four-dimensional structure (enhancement, social, coping and conformity motives of both music and alcohol use items) showed a poor fit to the data (Table 2). Only the eight-dimensional structure had a good fit (factor loadings, factor correlations, means and internal consistencies are provided in the Appendix).

**Table 2.** Model fit of the three confirmatory factor analyses estimated

	2-dimensional structure	4-dimensional structure	8-dimensional structure
CFI	.498	.544	.926
TLI	.438	.488	.909
RMSEA	.134	.128	.054
SRMR	.130	.134	.051

### Step 3: Structural equation modelling

Table 3 reveals that drinkers and non-drinkers differed in the links between music motives and health-related outcomes in adolescence. Almost all the links between conformity music motives and the health-related outcomes were significant for non-drinkers, whereas almost all the same links were not significant for drinkers. In this group, there was a significant negative link between enhancement music motives and being bullied, while the opposite was true for bullying others. There was also a significant positive link between enhancement music motives and evenings spent with friends among drinkers. With the exception of the latter, these links were in the same direction but were not significant among non-drinkers. Among non-drinkers, there was a significant negative link between enhancement music motives and depressed mood. Social music motives were related to both depressed mood and physical powerlessness. These links were also negative but not significant among drinkers. Almost all the effects of coping music motives on the health-related outcomes were significant among both drinkers and non-drinkers.

**Table 3.** Music motives as predictors of health indicators and social issues

Drinkers					Non-drinkers			
	Enh.	Social	Coping	Conf.	Enh.	Social	Coping	Conf.
Life satisfaction	.072*	.060*	–.181***	–.025	.073*	.085*	–.254***	–.091*
School pressure	.032	–.011	.115***	.025	–.065	.033	.120***	.065*
Self-rated health	–.001	.095***	–.116***	–.011	.013	.130***	–.175***	–.077*
Somatic complaints	–.002	.001	.139***	.050	–.058	–.014	.186***	.132*
Aggressive mood	.043	–.030	.164***	.002	–.051	–.032	.253***	.122*
Depressed mood	–.026	–.037	.232***	.059*	–.099*	–.072*	.244***	.225***
Physical powerlessness	.006	–.028	.195***	–.017	–.036	–.084*	.221***	.137*
Being bullied	–.075*	–.090*	.115***	.067*	–.042	–.092*	.149***	.034
Bullying others	.083*	.076*	–.019	.078*	.020	.102***	.002	.069*
Evenings with friends	.084*	.173***	–.026	.063*	–.013	.282***	.021	.078*

*Note:* Adjusted for gender and age effects; all coefficients are standardized regression weights (Beta); ^*^ *P* < 0.05; ^***^ *P* < 0.001

Almost all links between music motives and health-related outcomes in adolescence remained significant when drinking motives were included in the model (Table 4). There were also significant effects of drinking motives on outcomes in adolescence. However, for example, the same effects between enhancement music motives and the health-related outcomes in adolescence were significant with and without drinking motives.

**Table 4.** Music motives and drinking motives as predictors of health indicators and social issues

Music motives					Drinking motives			
	Enh.	Social	Coping	Conf.	Enh.	Social	Coping	Conf.
Life satisfaction	.069*	.068*	–.122***	–.007	–.003	–.017	–.249***	.020
School pressure	.022	–.016	.097***	.017	.059*	–.013	.083*	.002
Self-rated health	–.004	.108***	–.075*	.008	–.015	–.026	–.172***	–.007
Somatic complaints	–.005	–.009	.109***	.038	.034	.012	.127***	–.002
Aggressive mood	.047	–.033	.107***	–.016	.003	.003	.238***	–.010
Depressed mood	–.009	–.043	.164***	.022	–.052*	.037	.271***	.025
Physical powerlessness	.003	–.066*	.165***	–.029	.043	.073*	.133***	–.001
Being bullied	–.062*	–.071*	.095***	*.025*	–.033	–.035	.062*	.095*
Bullying others	.074*	*.031*	–.040	.074*	.073*	.078*	.102***	–.011
Evenings spent with friends	.079*	.105***	–.064*	.063*	.065*	.145***	.168***	–.039

*Note:* Adjusted for gender and age effects; all coefficients are standardized regression weights (Beta); * *P* < 0.05; *** *P* < 0.001; changes in the significance levels in comparison to the model without drinking motives (see column ‘Drinkers’ in Table 3) are in italic.

Certain links between music motives and health-related outcomes even became significant when drinking motives were taken into account, e.g. between social music motives and physical powerlessness, and between coping music motives and evenings spent with friends. Certain links disappeared when drinking motives were taken into account, e.g. between social music motives and bullying others, between conformity music motives and depressed mood, and between conformity music motives and being bullied.

Table 4 also shows that the significant links of music and drinking motives and health-related outcomes almost always went in the same direction (i.e. either both positive or both negative). Two exceptions were the effects of social music motives and social drinking motives on physical powerlessness, and the effects of coping music motives and coping drinking motives on evenings spent with friends. The link between social music motives and physical powerlessness was negative, whereas the one for social drinking motives was positive. The same is true for the link between coping music motives and evenings spent with friends (negative) and coping drinking motives and evenings spent with friends (positive).

## DISCUSSION

The purpose of this study was to investigate the links between music motives and health-related outcomes among non-drinkers and drinkers and whether these links are still significant among drinkers when drinking motives are taken into account.

### Differences in music motives between drinkers and non-drinkers

The results show that drinkers were less likely to indicate high enhancement music motives than non-drinkers. This is in contrast with research showing that individuals who drink for enhancement motives are sensation seekers (Kuntsche, Knibbe, Gmel & Engels, 2006), which served as a basis for the hypothesis that drinkers would score higher on enhancement music motives than non-drinkers. A possible explanation for the contrary finding could be that, because of its psy-choactive properties, alcohol has a stronger potential for seeking extreme sensations than listening to music. Due to its strong psycho-stimulant effects (e.g. heart rate acceleration) alcohol seems to be particularly suited to obtaining enhancing effects (Comeau, Stewart & Loba, 2001).

Drinkers were also less likely to indicate a higher level of conformity music motives than non-drinkers. Conformity-driven drinkers usually have the lowest level of endorsement of the four drinking motives (Kuntsche, Stewart & Cooper, 2008), i.e. most drinkers do not drink to be accepted by peers or to fit in with a group. Similarly, when it comes to motives for listening to music, research shows that listening to music to please friends and to gain popularity were the least indicated motives by adolescents (Tarrant, North & Hargreaves, 2000). Thus, because drinkers do not usually feel pressured by peers to drink alcohol, it is likely that they do not feel pressured by peers to listen to music either.

Drinkers were more likely to indicate high social music motives than non-drinkers. Research shows that heavy drinking among adolescents and young adults often happens in a social context. Just a small percentage of the adolescents drink heavily when alone (Christiansen, Vik & Jarchow, 2002; Kuntsche & Gmel, 2004). Because drinking usually happens in a social context, it is likely that drinkers also listen to music due to social motives.

### Testing the dimensionality of drinking motives and music motives

To confirm the theoretically assumed four-dimensional structure (Cooper, 1994; Cooper et al., 1998; Cox & Klinger, 1988, 1990), our second aim was to test whether drinking motives and music motives form eight distinct dimensions. Indeed, results from confirmatory factor analyses showed that the eight-factor model had a better fit than any alternative model with CFI, TLI, RMSEA and SRMR values that were close to recommended threshold (Schreiber et al., 2006) or better. This extends the findings of Kuntsche and Kuntsche (2009) and Kuntsche, Le Mével and Berson (in press) by demonstrating the existence of the four-factor structure of drinking motives and music motives when analysed separately but also when tested within the same study and questionnaire.

### Links between music motives and health-related outcomes in the presence of drinking motives

Our third aim was to test whether music motives are still significant predictors of health-related outcomes among drinkers when drinking motives are taken into account. First, differences in the links between music motives and health-related outcomes in adolescence between drinkers and non-drinkers were investigated. There was a significant link between conformity music motives and almost all the health-related outcomes among non-drinkers, whereas these links did not exist among drinkers. Research shows that individuals who drink for conformity motives are self-conscious and control their feelings of social awkwardness by drinking alcohol (Stewart & Devine, 2000) not by listening to music. If they do not drink alcohol, listening to music may play a stronger role in this respect but clearly more research is needed to gain further insights into the interplay between alcohol use, listening to music for conformity motives and health-related outcomes.

Moreover, the links between enhancement music motives and bullying others and between enhancement music motives and evenings spent with friends were significant among drinkers, but not among non-drinkers. Enhancement drinkers are often extraverted, sociable, excitement-seeking, and impulsive (Kuntsche et al., 2006). They are more likely to engage in aggressive behaviour like bullying others and to seek social company.

Another notable result is that almost all the links between coping music motives and the health-related outcomes are significant for both drinkers and non-drinkers. This may be explained by the link between coping motives and personality. Drinking to cope with negative feelings is associated with the personality trait neuroticism. Individuals that score high on this trait often experience negative feelings and may use different coping strategies to manage these feelings (Kuntsche et al., 2006). These coping strategies may be for example drinking alcohol or listening to music.

Subsequently, among alcohol-using adolescents, drinking motives were included. The results showed that almost all links remained significant between music motives and health-related outcomes in adolescence when drinking motives were taken into account and all the significant links were in the same direction. This can be explained by the importance of music in adolescence. As mentioned earlier, music can help adolescents with identity formation, peer affiliation, expressing agency and managing emotions (Laiho, 2004).

Although the effects of music motives and drinking motives on health-related outcomes were often in the same direction (i.e. either both positive or both negative), there were some notable exceptions in the links between social music/drinking motives and physical powerlessness, and between coping music/drinking motives and evenings spent with friends. The positive link between social drinking motives and physical powerlessness in contrast to the negative link between social music motives may be explained by the negative consequences of alcohol use. Alcohol use has a lot of negative (physical) consequences like being sick or having a hangover the day after, blackouts or getting involved in accidents (Gmel, Rehm & Kuntsche, 2003), whereas music does not have these consequences. The only direct physical negative consequence of listening to music can be hearing impairment, when individuals listen to music that is too loud (Daniel, 2007).

When it comes to the positive link between coping drinking motives and evenings spent with friends (the negative link for coping music motives), one explanation might be that drinking usually happens in a social context; solitary drinking is not usual (Christiansen et al., 2002; Kuntsche & Gmel, 2004). By contrast, listening to music may be done alone or together with peers (Tarrant et al., 2000). It appears that to cope with negative emotions, adolescents tend to listen to music on their own but drink together with peers.

Certain links between music motives and health-related outcomes disappeared when drinking motives were added. This was particularly the case for bullying. Violence is likely to occur in peer groups where heavy drinking is common (Kuntsche & Gmel, 2004). Adolescents who drink for conformity motives often act violently themselves (Kuntsche, 2007), probably because they want to avoid peer rejection from a group in which violence is common. Listening to music because of conformity motives does not appear to play a prominent role in this respect.

### Limitations

First, the results are representative of adolescents in Switzerland, but it remains unclear if the results can be generalized to other countries. Although drinking motives were found to be invariant across countries (Kuntsche et al., 2008; Kuntsche et al., 2014), there might be differences in music motives across cultures. Future research is needed to validate the MLMQ cross-nationally. A second limitation is the cross-sectional nature of the data, which makes it possible to demonstrate links between the different variables used in this study, but which makes it impossible to demonstrate cause-and-effect relationships. Future research is needed to investigate changes in both music motives and drinking motives over time.

### Implications for prevention

Personality-specific strategies have demonstrated, for example, that it is possible to reduce coping drinking motives by targeting anxiety-sensitivity (Conrod, Castellanos-Ryan & Mackie, 2011). Another effective way of reducing alcohol use is by offering functional alternatives (Correia, Simons, Carey & Borsari, 1998; Murphy, Colby, Correia & Vuchinich, 2005). For example, it may be promising to teach adolescents how to find relief and to cheer up by listening to music or how to have more fun with friends with music instead of using the psychoactive properties of alcohol for those purposes. Besides targeting specific drinking motives, prevention programs should therefore consider offering music as a functional alternative to alcohol use.

## CONCLUSIONS

This study showed important links between music motives and health-related outcomes among non-drinking adolescents and among drinkers even when drinking motives were taken into account. Music usually serves important functions in the lives of adolescents, even for those who use alcohol for different motives. This makes listening to music a promising candidate to serve as a functional alternative to alcohol use. Furthermore, music motives and drinking motives were shown to form eight distinct dimensions, pointing to the potential to attenuate one behaviour (drinking alcohol) by reinforcing another (listening to music). This is particularly important because listening to music usually has fewer negative consequences than excessive alcohol consumption (Daniel, 2007; Gmel et al., 2003). However, despite the fact that the function that music serves for a given individual heavily depends on personal taste, including music in prevention efforts also means that one has to pay attention to the kind of music. For example, alcohol-related lyrics in songs may instigate alcohol consumption (Engels et al., 2011). Moreover, a personal preference for certain musical genres such as house, techno, rock, heavy metal, punk and gothic has been found to be associated positively with substance use (ter Bogt et al., 2012).

## Figures and Tables

**Table:** Results of the Confirmatory Factor Analysis to test the eight-dimensional structure of drinking motives and music motives

*When you think about all the times you drank alcohol* *(beer, wine, spirits, etc.) over the past 12 months, how many times did you do it …*	Drinking motives				Music motives			
	Enhancement	Social	Coping	Conformity	Enhancement	Social	Coping	Conformity
because you like the feeling?	.837							
to get high?	.786							
because it is fun?	.688							
because it helps you to enjoy a party?		.778						
because it makes social gatherings more fun?		.776						
because it improves parties and celebrations?		.822						
because it helps you when you feel depressed or nervous?			.883					
to cheer you up when you are in a bad mood?			.818					
to forget about your problems?			.810					
to be liked?				.730				
to fit in with a group you like?				.879				
so you won’t feel left out?				.798				
*How often do you listen to music for the following reasons…*								
because you like the feeling?					.737			
to get high?					.477			
because it is fun?					.573			
because it helps you to enjoy a party?						.828		
because it makes social gatherings more fun?						.569		
because it improves parties and celebrations?						.844		
because it helps you when you feel depressed or nervous?							.894	
to cheer you up when you are in a bad mood?							.890	
to forget about your problems?							.689	
to be liked?								.741
to fit in with a group you like?								.904
so you won’t feel left out?								.852
Correlation with the factor “Drinking Social”	.813							
Correlation with the factor “Drinking Coping”	.492	.399						
Correlation with the factor “Drinking Conformity”	.291	.260	.365					
Correlation with the factor “Music Enhancement”	.297	.225	.126	.015				
Correlation with the factor “Music Social”	.270	.461	.101	.038	.625			
Correlation with the factor “Music Coping”	.076	.110	.252	.048	.657	.416		
Correlation with the factor “Music Conformity”	.079	.085	.159	.415	.240	.154	.163	
Means *(SD)*	2.10	2.33	1.49	1.15	3.14	3.23	3.23	1.27
	(1.12)	(1.20)	(0.86)	(0.48)	(0.98)	(1.31)	(1.30)	(0.70)
Internal consistencies (Cronbach’s alpha)	.835	.867	.878	.836	.607	.815	.861	.827

*Note:* All factor loadings are standardized item loadings and are significant at the 0.01 % level.
